# Association between *CYP3A5* Polymorphism and Statin-Induced Adverse Events: A Systemic Review and Meta-Analysis

**DOI:** 10.3390/jpm11070677

**Published:** 2021-07-19

**Authors:** Jeong Yee, Hamin Kim, Yunhee Heo, Ha-Young Yoon, Gonjin Song, Hye-Sun Gwak

**Affiliations:** College of Pharmacy and Graduate School of Pharmaceutical Sciences, Ewha Womans University, Seoul 03760, Korea; jjjhello1@naver.com (J.Y.); mamin94@ewhain.net (H.K.); heo2119@naver.com (Y.H.); hayoungdymphnayoon@gmail.com (H.-Y.Y.); songgonjin@naver.com (G.S.)

**Keywords:** statin, adverse event, CYP3A5*3, pharmacogenomics, systematic review, meta-analysis

## Abstract

**Purpose:** Cytochrome P450 (CYP) is involved in the metabolism of statins; CYP3A5 is the main enzyme responsible for lipophilic statin metabolism. However, the evidence of the association between CYP3A5*3 polymorphism and the risk of statin-induced adverse events remains unclear. Therefore, this study aimed to perform a systematic review and meta-analysis to investigate the relationship between the CYP3A5*3 polymorphism and the risk of statin-induced adverse events. **Methods:** The PubMed, Web of Science, and EMBASE databases were searched for qualified studies published until August 2020. Observational studies that included the association between statin-induced adverse events and the CYP3A5*3 polymorphism were reviewed. The odds ratios (ORs) and 95% confidence intervals (CIs) were evaluated to assess the strength of the relationship. The Mantel–Haenszel method was used to provide the pooled ORs. Heterogeneity was estimated with I^2^ statistics and publication bias was determined by Begg’s and Egger’s test of the funnel plot. Data analysis was performed using Review Manager (version 5.4) and R Studio (version 3.6). **Results:** In total, data from 8 studies involving 1614 patients were included in this meta-analysis. The CYP3A5*3 polymorphism was found to be associated with the risk of statin-induced adverse events (*3/*3 vs. *1/*1 + *1/*3: OR = 1.40, 95% CI = 1.08–1.82). For myopathy, the pooled OR was 1.30 (95% CI: 0.96–1.75). The subgroup analysis of statin-induced myopathy revealed a trend, which did not achieve statistical significance. **Conclusions:** This meta-analysis demonstrated that the CYP3A5*3 polymorphism affected statin-induced adverse event risk. Therefore, *CYP3A5* genotyping may be useful to predict statin toxicity.

## 1. Introduction

Statins, 3-hydroxy-3-methylglutaryl coenzyme A (HMG-CoA) reductase inhibitors (Supplementary Materials Figure S1), are one of the most prescribed medications for both primary and secondary prevention of atherosclerotic cardiovascular events [1]. These drugs decrease cholesterol synthesis by competitively inhibiting HMG-CoA reductase, which catalyzes the conversion of HMG-CoA to mevalonate, a precursor of cholesterol [2].

The most common adverse events of statins include constipation, abdominal pain, nausea, dyspepsia, muscle pain, headache, and skin rash [3]. Although statins are generally safe and well-tolerated, some adverse events, especially muscle-related symptoms and liver toxicity, may cause statin intolerance. According to Mancini et al., almost 20–30% of patients have experienced statin intolerance [4]. As statin discontinuation can increase the risk of acute cardiovascular risk, statin adverse events and intolerance should be managed appropriately [5,6].

Advanced age, female sex, Asian ethnicity, pre-existing liver and kidney diseases, high-dose statin therapy, and drug interactions are known to be risk factors for statin intolerance [7]. In addition, recent studies have reported the pharmacogenetic features in relation to statin adverse events [8,9]. Most studies have focused on the association between the solute carrier organic anion transporter family 1B1 (*SLCO1B1)* gene and risk of statin-induced adverse events [10,11]. The Clinical Pharmacogenetics Implementation Consortium (CPIC) provided clinical recommendations for simvastatin-induced myopathy based on *SLCO1B1* genotype [12]. Nevertheless, other genetic factors still remain to explain statin-induced adverse events.

As most statins are primarily metabolized by two members of the cytochrome P450 superfamily, CYP3A4 and CYP3A5 [13] (Supplementary Materials Figure S2), and CYP3A5*3 is one of the functionally important single-nucleotide polymorphisms (SNPs) with high allele frequency (5%, 22%, 28%, and 81% in European, American, Asian, and African populations, respectively) [14,15], it is reasonable to assume that CYP3A5*3 may influence the risk of statin-induced adverse events. Therefore, we conducted a systematic review and meta-analysis to assess the genetic association between the CYP3A5*3 polymorphism and the risk of statin-induced adverse events.

## 2. Methods and Materials

### 2.1. Literature Search Strategy and Inclusion Criteria

This meta-analysis was conducted according to the checklist outlined in the Preferred Reporting Items for Systematic Reviews and Meta-Analyses [16]. Two reviewers independently searched for studies that had been published until August 11, 2020. An extensive search of electronic databases (PubMed, Web of Science, and EMBASE) was performed using the following search terms: (statin* OR (3-hydroxy-3-methylglutaryl coenzyme A reductase inhibitor*) OR (hydroxymethylglutaryl coenzyme A reductase inhibitor*) OR (HMG-CoA reductase inhibitor*) OR atorvastatin OR cerivastatin OR crilvastatin OR fluvastatin OR lovastatin OR mevastatin OR pitavastatin OR pravastatin OR rosuvastatin OR simvastatin) AND (CYP3A5* OR (cytochrome p450 3A5) OR (cytochrome p450 3A5)) AND (polymorph* OR variant* OR mutation* OR genotyp* OR phenotyp* OR haplotyp* OR allele* OR SNP* OR pharmacogen*). There was no language limitation.

Studies were included if (i) they evaluated the association of the CYP3A5*3 polymorphism with statin-induced adverse events; (ii) they were based on observational design (cohort study, case-control, or cross-sectional study); and (iii) they provided sufficient information to calculate odds ratios (ORs) and 95% confidence intervals (CIs). Studies were excluded if they were (i) not original articles (e.g., conference/meeting abstract, reviews, comments, letters, editorials, or protocols); (ii) in vitro or animal studies; or (iii) studies on healthy subjects. In case of overlapping data, only the most recent and comprehensive data were included in the meta-analysis.

### 2.2. Data Extraction and Study Quality Assessment

Two reviewers independently extracted data, and discrepancies were resolved by consensus. Extracted data included the following information: the name of the first author, publication year, country, study design, number of cases and controls, mean age, percentage of males, case definition, statins included in the analysis, and the genotyping method. Articles were assessed by two investigators based on the Newcastle–Ottawa Scale (NOS) evaluating studies in the three categories of selection, comparability, and outcome assessment [17]. The score range of NOS is from 0 to 9.

### 2.3. Statistical Analysis

The meta-analysis was performed using Review Manager (version 5.4; The Cochrane Collaboration, Copenhagen, Denmark). ORs and 95% CIs were used to identify the relationship between the CYP3A5*3 polymorphism and the risk of statin-induced adverse events. The Mantel–Haenszel method and *Z*-test were used to evaluate the significance of the pooled OR [18]. A P-value < 0.05 was considered statistically significant. The heterogeneity across studies was estimated by way of a chi-square test and an *I^2^* statistic. Given the high heterogeneity (I^2^ > 75%), a random-effects model was applied [19].

Subgroup analysis was performed in patients with statin-induced myopathy. Both Begg’s rank correlation test and Egger’s regression test of the funnel plot were performed using R Studio software (version 3.6.0; R Foundation for Statistical Computing, Vienna, Austria) to detect publication bias [20,21]. To evaluate the robustness of the results, sensitivity analysis was conducted by sequentially omitting each study.

## 3. Results

### Literature Search

**Figure 1:**
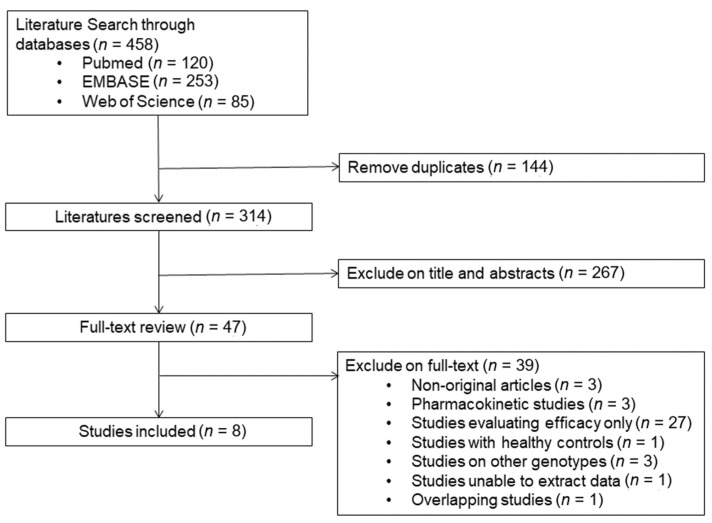
Flow diagram of study selection.

A detailed flow chart of the study selection process is presented in Figure 1. A total of 458 studies were identified from searches of three databases. After removal of 144 duplicates, 314 records were initially identified, of which the titles and abstracts were screened for inclusion in this study. From this initial review, 47 studies were selected for full-text reviews and assessed for eligibility. Of these 47 studies, 39 were excluded for the following reasons: non-original articles (*n* = 3), pharmacokinetic studies (*n* = 3), studies evaluating efficacy only (*n* = 27), studies with healthy control groups (*n* = 1), studies on other genotypes (*n* = 3), studies unable to extract data (*n* = 1), and overlapping studies (*n* = 1). Ultimately, eight articles were selected for this systematic review [22,23,24,25,26,27,28,29].

The characteristics of the included studies are presented in Table 1. The studies were published from 2005 to 2018, and all studies were conducted on adults. Four of them were conducted in Asia, three in America, and one in Europe. There were two cohort studies and six case-control studies, with NOS scores ranging from 6 to 9.

**Figure 2:**
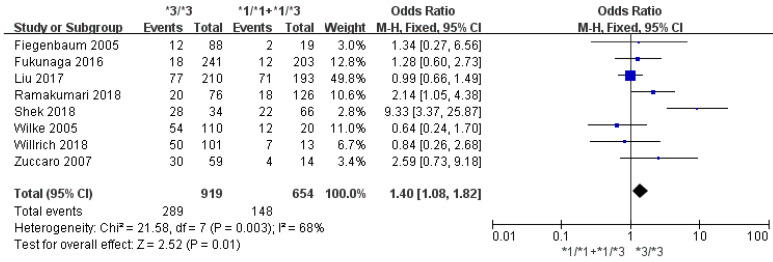
Forest plots of the association between CYP3A5*3 and statin adverse events.

**Figure 3:**
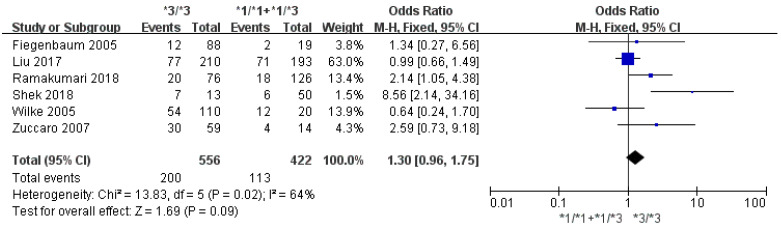
Forest plots of the association between CYP3A5*3 and statin-induced myopathy.

Eight studies involving 1641 patients were evaluated to investigate the association between the CYP3A5*3 polymorphism and the risk of statin-induced adverse events. A statistically significant association was found between the CYP3A5*3 polymorphism and the risk of statin-induced adverse events (*3/*3 vs. *1/*1 + *1/*3: OR = 1.40, 95% CI = 1.08–1.82; I^2^ = 68%; Figure 2). In a subgroup analysis for statin-induced myopathy, the CYP3A5*3 polymorphism showed a similar trend; patients with the CYP3A5*3/*3 showed a higher risk of myopathy after statin treatment compared to the CYP3A5*3 allele carriers, although it did not achieve a statistical significance (*3/*3 vs. *1/*1 + *1/*3; OR = 1.30; 95% CI = 0.96–1.75; I^2^ = 64%; Figure 3). The results of Begg’s and Egger’s test did not reveal any publication bias for statin-induced adverse events (*p* = 0.4579 and *p* = 0.5325, respectively) and myopathy (*p* = 0.3476 and *p* = 0.2251, respectively) (Supplementary Materials Figure S3). The sensitivity analysis by omitting each study showed mostly similar results to that from the entire meta-analysis (OR range: 1.17–1.80). The result was stable except for excluding Shek et al. [27].

## 4. Discussion

In summary, our results showed that the CYP3A5*3 polymorphism increased the risk of statin-induced adverse events. Subgroup analysis showed that CYP3A5*3 tended to increase the risk of statin-induced myopathy without statistical significance, possibly because of insufficient power due to the small sample size. No significant publication bias was detected, and sensitivity analysis provided stable results except for excluding Shek et al. [27]. As the OR of CYP3A5*3 and statin-induced adverse event risk was high in the study of Shek et al. [27] and this study might largely influence the pooled data, the findings in the meta-analysis should be interpreted carefully.

Statins can induce a wide range of adverse events from mild gastrointestinal symptoms to autoimmune disorders; however, statin-induced adverse events are often dose dependent [30,31]. Most studies included in this meta-analysis investigated myopathy; the pooled estimate of the effects of CYP3A5*3 on myopathy was similar to the overall one, although it showed a marginally statistical significance. The failure to achieve statistical significance in the subgroup analysis with myopathy was attributable to two studies, which had opposite trends [24,28]. There are two studies investigating the association between CYP3A5*3 and liver injury. A study by Shek et al. [27] revealed that the CYP3A5*3 polymorphism increased the risk of hepatotoxicity (OR 9.63, 95% CI: 3.29–28.1), whereas a study by Fukunaga et al. [23] did not find significant differences (OR 1.28, 95% CI: 0.60–2.73).

Several studies investigated the effect of CYP3A inhibitors (e.g., amiodarone, itraconazole, ritonavir) on the adverse events of statins [32,33]. Similarly, grapefruit juice, which contains inhibitors of CYP3A, triggered statin-induced rhabdomyolysis [34]. The aforementioned studies suggest that the CYP3A enzyme played an important role in statin metabolisms. CYP3A5 is responsible for at least 50% of the total hepatic CYP3A content. As CYP3A5*3 is the most prevalent variant allele related to reduced enzyme activity by alternative splicing and protein truncation of the CYP3A5 protein [35], the *CYP3A5* gene could be the leading genetic contributor to inter-individual differences in CYP3A-dependent drug clearance and responses.

Several meta-analyses have been performed to examine the association between CYP3A5*3 polymorphism and drug responses. A meta-analysis on the association between CYP3A5*3 and tacrolimus response in kidney transplant recipients revealed that CYP3A5*1 allele carriers had a higher risk of acute rejection and chronic nephrotoxicity than CYP3A5*3/*3 carriers. The plasma concentration divided by the daily dose per body weight was significantly lower among CYP3A5*1 allele carriers compared with CYP3A5*3/*3 carriers [36]. Similar results were found in other meta-analyses in renal transplant recipients treated with cyclosporine; CYP3A5*3 polymorphism was found to be associated with the cyclosporine dose-adjusted concentration. Patients carrying the CYP3A5*3/*3 genotype required a lower dose of cyclosporine to reach target levels than CYP3A5*1 allele carriers [37]

Lipophilic statins, such as atorvastatin, lovastatin, and simvastatin, are known to be metabolized primarily by CYP3A [23]. In line with our results, it was reported that CYP3A5*3/*3 was associated with decreased statin clearance, along with increased statin exposure and decreased clearance [38]. In addition, a study on the pharmacodynamic effects of CYP3A5*3 reported that CYP3A5*1 carriers had significantly less effective responses than those with CYP3A5*3/*3 during lipophilic statin therapy [39].

Studies by Liu [24] and Ramakumari [26] included not only lipophilic agents, but also hydrophilic agents. However, we included those studies because in the study by Liu, atorvastatin and simvastatin comprised more than 70% of the statins administered, while in the study by Ramakumari on patients with atorvastatin and rosuvastatin in a 40:60 ratio, it was found that the CYP3A5*3 polymorphism significantly increased statin-induced myopathy. With respect to the association between rosuvastatin and CYP3A5, the Genetic Effects On STATins (GEOSTAT-1) study reported a significantly enhanced response to rosuvastatin in patients with variant genotypes of CYP3A5 [40].

In addition to genetic factors, several factors can affect drug metabolism, such as age, sex, nutritional conditions, comorbidities, and concomitant medications [41]. Such non-genetic factors can cause phenoconversion, which is the mismatch between the genotype-based prediction of drug metabolism and true metabolizing capacity [42]. Therefore, the effects of genetic polymorphisms should be interpreted in line with the patients’ clinical features [43].

This study has some limitations. First, the number of studies was relatively small, making it difficult to achieve sufficient statistical power for subgroup analysis. Second, there was clinical heterogeneity, including the definition of adverse events. Third, we could not adjust the statin type, dose, or the concomitant medications, which could affect the occurrence of statin-induced adverse events. Despite those limitations, to our knowledge, this is the first meta-analysis to reveal an association between the CYP3A5*3 polymorphism and the risk of statin-induced adverse events.

## 5. Conclusions

This study showed that the CYP3A5*3 polymorphism increased the statin-induced adverse event risk. This finding suggests that *CYP3A5* genotyping can help individualized statin therapy. Further studies are needed to evaluate the utility of *CYP3A5* genotyping in statin therapy.

## Figures and Tables

**Table 1:** Characteristics of the studies included.

Study	Country	Study Design	Case/Control Number	Mean Age of Case/Control	Male % of Case/Control	Case Definition	Statins Included in the Analysis	Genotyping Method	NOS
Fiegenbaum et al. (2005)	Brazil	Prospective cohort study	15/99	63.0/59.2	18.8/25.3	Myopathy	Simvastatin	PCR-RFLP	9
Fukunaga et al. (2016)	Japan	Case-control study	30/414	61.0/66.0 ^a^	60.0/53.9	Liver injury	Atorvastatin	Invader Assay	6
Liu et al. (2017)	China	Case-control study	148/255	60.6/63.3	82.4/82.7	Myopathy	Atorvastatin, fluvastatin, pravastatin, rosuvastatin, simvastatin	TaqMan	9
Ramakumari et al. (2018)	India	Retrospective cohort study	38/164	63.0 ^b^	64.9 ^b^	Myopathy symptoms with elevated CK level	Atorvastatin, rosuvastatin	PCR	7
Shek et al. (2017)	Uzbekistan	Case-control study	50/50	59.3/61.7	42.0/56.0	Elevated transaminase or CK level	Simvastatin	PCR-RFLP	6
Wilke et al. (2005)	USA	Case-control study	68/69	58.1/63.1	79.4/50.7	Myopathy symptoms with elevated CK level	Atorvastatin	Invader Assay	6
Willrich et al. (2018)	USA	Case-control study	57/57	65.5/65.7	56.6/56.6	Statin intolerance	Atorvastatin, lovastatin, simvastatin	TaqMan	9
Zuccaro et al. (2007)	Italy	Retrospective cohort study	50/50	61.4/61.1	44.0/46.0	Myopathy	Atorvastatin, simvastatin.	PCR-RFLP	8

CK: creatine kinase; NOS: Newcastle–Ottawa Scale; PCR: polymerase chain reaction; RFLP: restriction fragment length polymorphism.^a^ Expressed as the median ^b^ Data for all participants.

## Data Availability

No new data were created or analyzed in this study. Data sharing is not applicable to this article.

## Supplementary Materials

The following are available online at https://www.mdpi.com/article/10.3390/jpm11070677/s1, Figure S1. Chemical structures of statins. The chemical structure for each drug was drawn with the PubChem Sketcher V2.4. (a) Atorvastatin. (b) Fluvastatin. (c) Lovastatin. (d) Pravastatin. (e) Rosuvastatin. (f) Simvastatin. Figure S2. CYP3A4/5 metabolic pathways of statins. Figure S3. Funnel plot of the association between CYP3A5*3 and statin-induced adverse events. (a) Statin adverse events. (b) Statin-induced myopathy.

## Abbreviations

CI	confidence interval
CK	creatine kinase
CPIC	Clinical Pharmacogenetics Implementation Consortium
CYP	cytochrome P450
GEOSTAT-1	Ge-netic Effects On STATins
HMG-CoA	3-hydroxy-3-methylglutaryl coenzyme A
NOS	Newcastle–Ottawa Scale
OR	odds ratio
PCR	polymerase chain reaction
RFLP	re-striction fragment length polymorphism
SLCO1B1	solute carrier organic anion transporter family 1B1
SNP	single-nucleotide polymorphism
