# Supplementary material for: Association between CYP3A5 Polymorphism and Statin-Induced Adverse Events: A Systemic Review and Meta-Analysis
Source: J Pers Med. 2021 Jul 19;11(7):677. doi: 10.3390/jpm11070677 (PMC8304457; doi:10.3390/jpm11070677)
Supplement: Supplementary file 1 [file jpm-11-00677-s001.zip › jpm-1293049-supplementary.pdf]

(a)

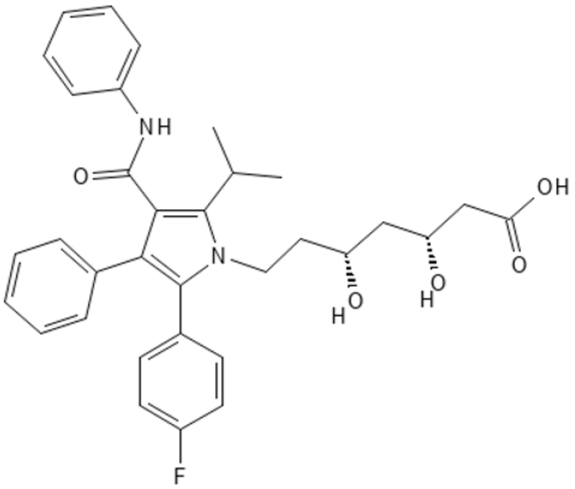

(b)

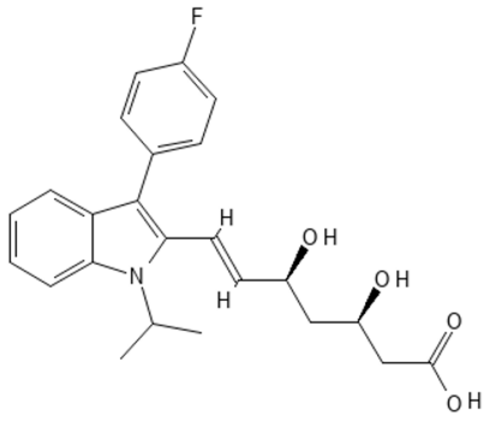

(c)

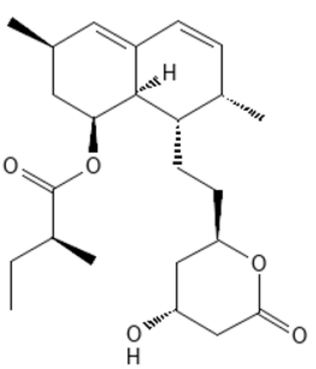

(d)

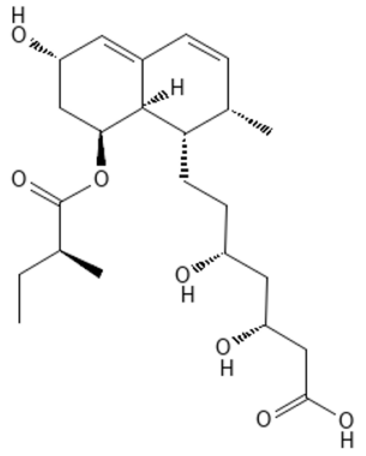

(e)

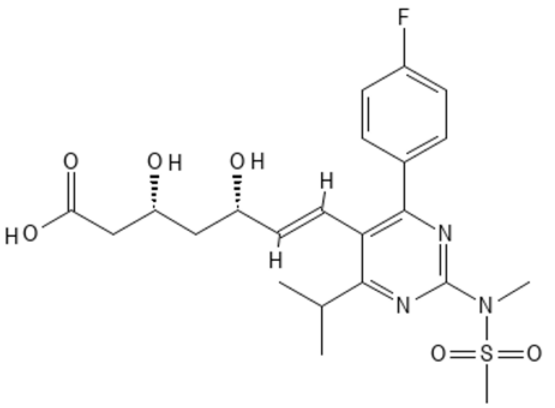

(f)

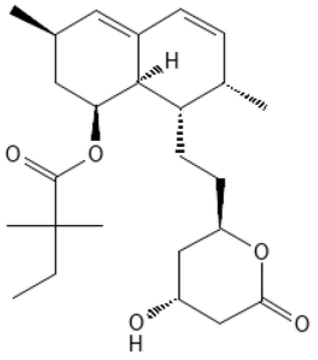

Supplementary Figure S1. Chemical structures of statins. The chemical structure for each drug was drawn with the PubChem Sketcher V2.4. (a) Atorvastatin. (b) Fluvastatin. (c) Lovastatin. (d) Pravastatin. (e) Rosuvastatin. (f) Simvastatin.

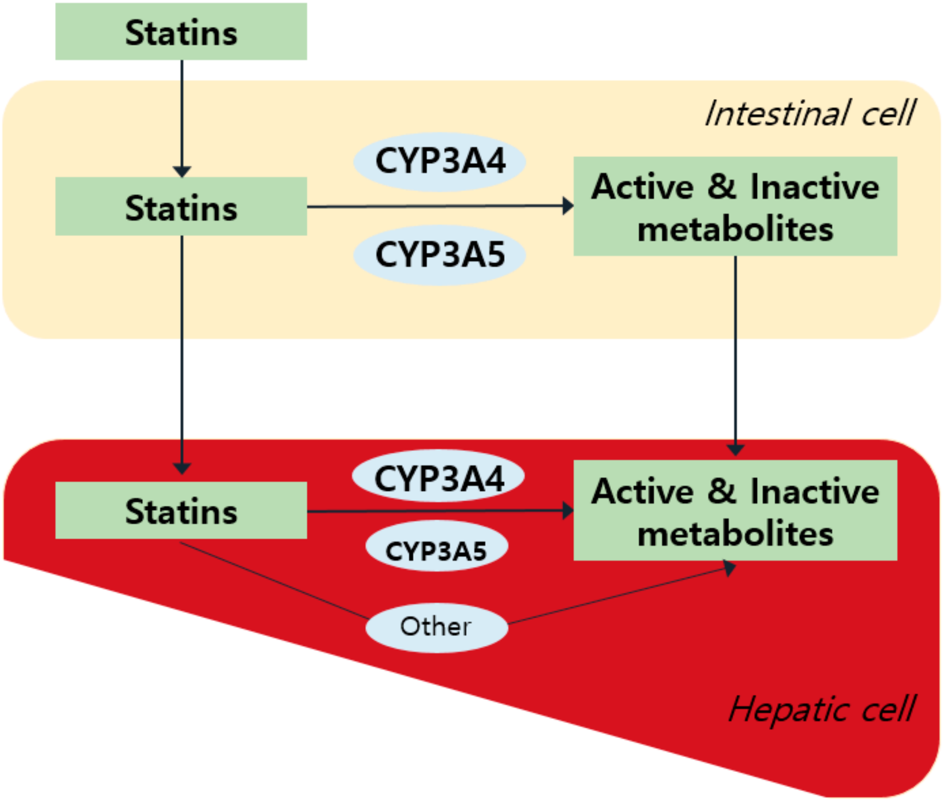

Supplementary Figure S2. CYP3A4/5 metabolic pathways of statins.

(a)

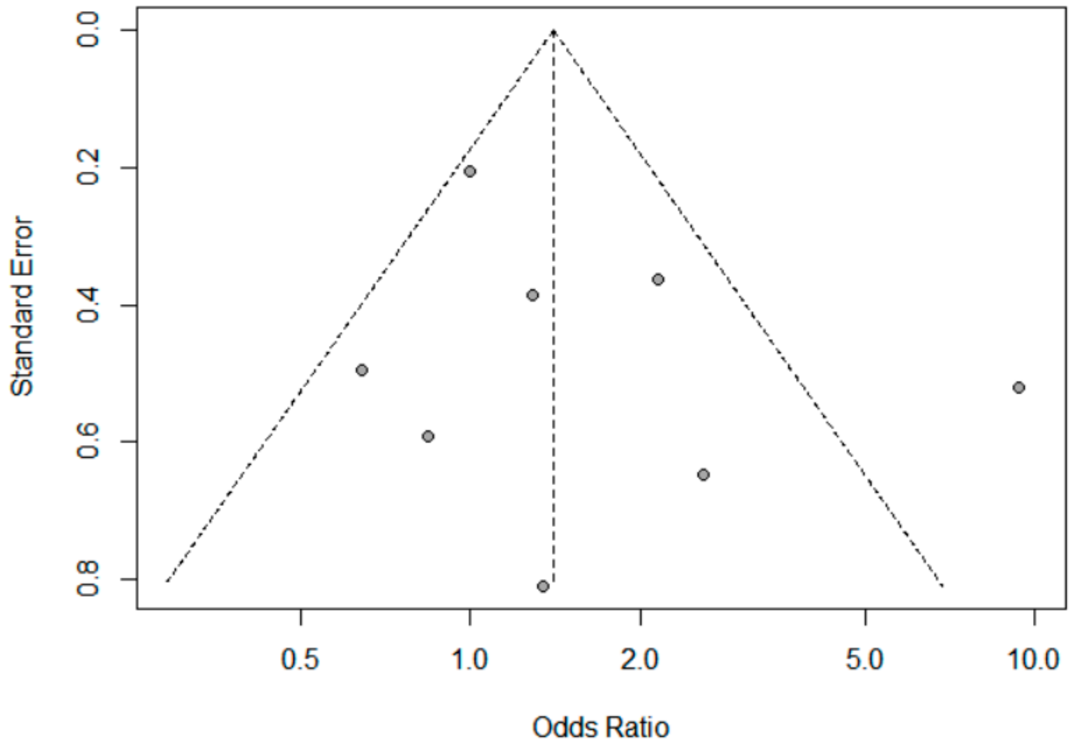

(b)

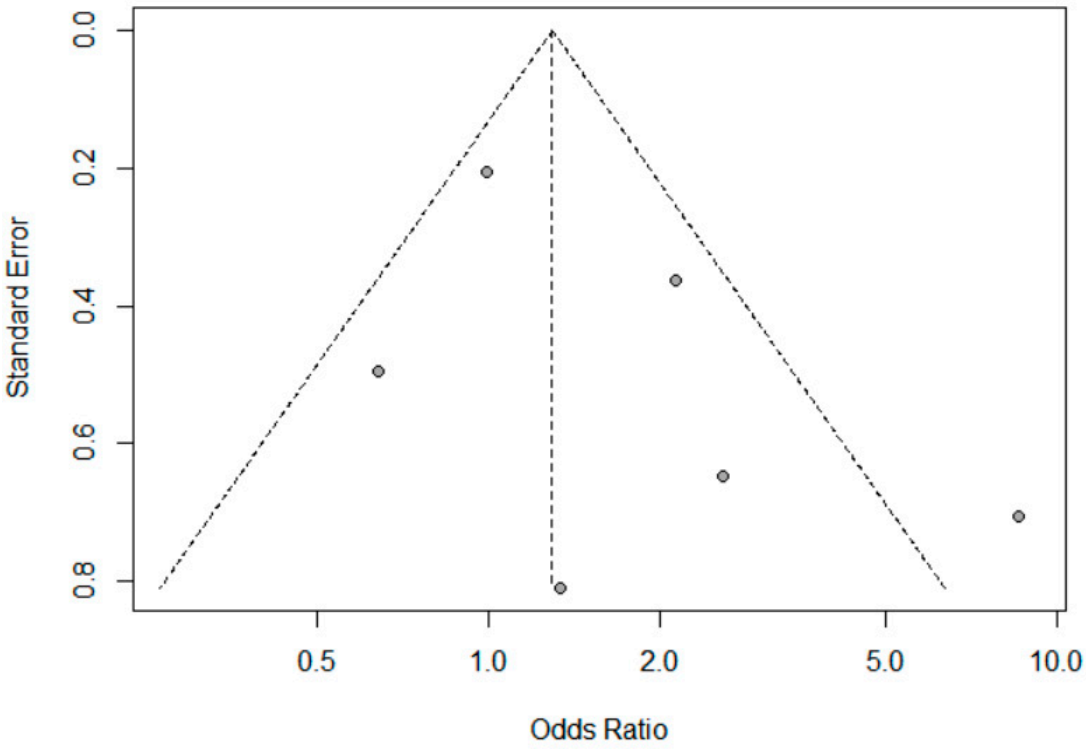

Supplementary Figure S3. Funnel plot of the association between *CYP3A5*3* and statin-induced adverse events. (a) Statin adverse events. (b) Statin-induced myopathy
